# Massachusetts State Veterinary Medical Association

**Published:** 1895-05

**Authors:** 


### MASSACHUSETTS STATE VETERINARY MEDICAL ASSOCIATION.

The annual meeting of the Massachusetts State Veterinary Medical Association elected the following officers for the ensuing year: *President*, J. M. Parker, Haverhill, Mass.; *Vice-Presidents*, C. Winslow, Rockland, Mass., W. A. Sherman, Lowell, Mass.; *Secretary-Treasurer*, H. P. Rogers, Alston, Mass.; *Executive Committee*: Drs. Thos. Blackwood, Chairman, Boston; J. F. Winchester, Lawrence; L. H. Howard, Boston; F. H. Osgood, Boston; E. C. Beckett, Boston.

